# Creatine Ameliorates the Adverse Effects of High-Fat Diet on Hepatic Lipid Metabolism *via* Activating Mfn2-Mediated Mitochondrial Fusion in Juvenile Grass Carp

**DOI:** 10.1155/anu/1151656

**Published:** 2025-05-08

**Authors:** Nan-Jun Hu, Guang-Li Feng, Xiao-Hong Lai, Mo Peng, Yu-Feng Song

**Affiliations:** ^1^Key Laboratory of Freshwater Animal Breeding, Ministry of Agriculture, Fishery College, Huazhong Agricultural University, Wuhan 430070, China; ^2^College of Animal Science and Technology, Jiangxi Agricultural University, Nanchang 330045, China

**Keywords:** creatine, grass carp, hepatic lipid metabolism, high-fat diet, mitochondrial fusion

## Abstract

With the increasing prevalence of high-fat diets (HFD) in aquaculture practices, the detrimental effects of HFD on farmed fish have garnered significant attention. Creatine has emerged as a promising green feed additive for aquaculture species; however, its potential role in mitigating the negative impacts of HFD remains poorly understood. To address this knowledge gap, the present study was designed to investigate the protective effects of dietary creatine supplementation on HFD-induced hepatic lipid metabolism disorders and muscle quality deterioration in juvenile grass carp (*Ctenopharyngodon idella*). Three experimental diets were formulated: a control diet (5.20% lipid, control), a HFD (8.11% lipid, HFD), and a HFD supplemented with 2% creatine (HFD + creatine). Juvenile grass carp (initial weight: 4.12 ± 0.02 g) were randomly allocated into nine 300-L indoor tanks and fed the experimental diets for 8 weeks. The key findings of this study revealed that (1) Dietary creatine supplementation significantly ameliorated the adverse effects of HFD on growth performance and feed utilization efficiency in juvenile grass carp. (2) Creatine supplementation improved muscle quality parameters in juvenile grass carp. (3) Dietary creatine attenuated HFD-induced hepatic lipid accumulation through enhanced fatty acid *β*-oxidation, which was mediated by mfn2-dependent mitochondrial fusion. Notably, this study elucidates a novel molecular mechanism whereby creatine activates mitochondrial fusion through the binding of *pparα* transcription factor to specific sites on the mitofusin 2 (Mfn2) gene promoter. To our knowledge, this is the first comprehensive investigation from a multi-organ/tissue perspective combined with mitochondrial dynamics analysis, providing valuable insights for developing effective nutritional strategies to counteract HFD-induced adverse effects in farmed fish through creatine supplementation.

## 1. Introduction

Dietary lipids are crucial in fish nutrition, serving as a primary energy source and supplying essential fatty acids that significantly influence growth and performance [1–3]. The protein-sparing effect, achieved by elevating lipid levels while reducing protein content, effectively decreases nitrogen emissions [4–6]. Consequently, the aquaculture industry often employs high-fat diets (HFD) to enhance economic efficiency and minimize nitrogen waste [7]. However, emerging evidence highlights the detrimental health impacts of HFD on fish [8–10]. Research has consistently shown that HFD leads to abnormal lipid accumulation in visceral and hepatic tissues, disrupting metabolic processes and impairing growth performance, which can escalate to increased mortality rates [11–13]. Therefore, developing effective nutritional strategies to counteract the adverse effects of HFD on hepatic lipid metabolism in fish is imperative. The growing concern over the negative health impacts of excessive HFD use in aquaculture has spurred interest in functional green feed additives [14]. Creatine, a non-proteinogenic organic acid, is vital in tissues with high energy demands [15, 16]. Previous studies have demonstrated that dietary creatine supplementation can enhance both growth and physical performance in farmed fish [17], positioning it as a promising alternative to antibiotics in fish diets [18]. Additionally, creatine has been shown to improve energy metabolism in adipocytes [15]. Given its role in energy metabolism, creatine may also mitigate lipid accumulation by enhancing energy utilization. Supporting this, a study in mice revealed that creatine regulates energy metabolism in beige and brown adipose tissues, reducing diet-induced lipid accumulation [19]. Despite these findings, the mechanisms by which creatine influences growth performance and its potential to alleviate HFD-induced hepatic lipid metabolic disorders remain poorly understood. Further research is necessary to elucidate these aspects and optimize the use of creatine in aquaculture nutrition.

In general, hepatic lipid accumulation results from the balance between *de novo* synthesis of fatty acids (lipogenesis) and lipid catabolism *via* lipolysis and mitochondrial *β*-oxidation, and many key enzymes and transcription factors are involved in the process [20]. Lipid catabolism through mitochondrial *β*-oxidation is the primary way of generating energy among these. The turnover of adenosine triphosphate (ATP), the energy source of the cell, is greatly facilitated by creatine in tissues [21]. Thus, the level of creatine closed is correlated with mitochondrial *β*-oxidation, and *β*-oxidation is responsible for maintaining energy homeostasis. In grass carp, dietary creatine has been shown to decrease lipid accumulation by improving mitochondrial *β*-oxidation in previous studies [22],, but further mechanistic investigations are required to confirm this. On the other hand, maintaining mitochondrial integrity and homeostasis is crucial because the mitochondrial matrix is the primary location for fatty acid (FA) *β*-oxidation [23]. Mitochondria are double-membrane organelles that form a highly dynamic network and undergo continuous cycles of fusion and fission [24]. These processes are essential for maintaining mitochondrial integrity, homeostasis, and function [25, 26]. Therefore, mitochondrial dynamics play a critical role in hepatic lipid metabolism, particularly in *β*-oxidation [27]. However, the molecular details regarding whether and how dietary creatine has an impact on mitochondrial *β*-oxidation though mitochondrial dynamics are still unknown.

Hepatic lipid accumulation is primarily governed by the equilibrium between *de novo* fatty acid synthesis (lipogenesis) and lipid catabolism through lipolysis and mitochondrial *β*-oxidation, with numerous key enzymes and transcription factors participating in this regulatory process [20]. Among these metabolic pathways, mitochondrial *β*-oxidation serves as the predominant mechanism for energy production. The cellular energy currency, adenosine triphosphate (ATP), is significantly influenced by creatine levels in tissues, which facilitate ATP turnover [21]. Consequently, creatine concentration is closely associated with mitochondrial *β*-oxidation activity, a critical process for sustaining energy homeostasis. Previous investigations in grass carp have demonstrated that dietary creatine supplementation reduces lipid accumulation through enhanced mitochondrial *β*-oxidation [22], although the underlying mechanisms warrant further elucidation. The maintenance of mitochondrial integrity and homeostasis is particularly crucial as the mitochondrial matrix serves as the principal site for FA *β*-oxidation [23]. Mitochondria, as double-membrane-bound organelles, constitute a highly dynamic network that undergoes continuous cycles of fusion and fission [24]. These dynamic processes are essential for preserving mitochondrial structural integrity, functional homeostasis, and overall cellular metabolism [25, 26]. Therefore, mitochondrial dynamics play a pivotal role in regulating hepatic lipid metabolism, particularly in the context of *β*-oxidation [27]. Nevertheless, the molecular mechanisms through which dietary creatine may influence mitochondrial *β*-oxidation *via* modulation of mitochondrial dynamics remain to be fully elucidated.

In China's aquaculture industry, grass carp (*Ctenopharyngodon idella*) represents the most extensively farmed freshwater fish species globally, with an annual production reaching 5,904,805 tons [28]. The sector has witnessed a growing trend of incorporating high-energy non-protein sources, particularly lipids, to enhance growth performance in aquaculture practices [29]. However, this approach has led to significant challenges, as grass carp fed with relatively high-fat commercial feeds demonstrate a propensity for excessive lipid accumulation in the mesentery and hepatopancreas during farming operations [30]. The nutritional requirements of grass carp include creatine, a crucial compound predominantly found in fish meal and other animal protein sources. Current evidence suggests that grass carp may encounter difficulties in obtaining sufficient creatine from plant protein-based diets [31], potentially limiting the performance of commercial feeds in the industry due to their relatively low creatine content. This study investigates the potential of creatine to suppress hepatic lipid aggregation in grass carp through the activation of mitochondrial dynamics, building upon similar findings observed in mammalian systems. To comprehensively address this hypothesis, we conducted both in vivo and in vitro experiments following creatine treatment, measuring hepatic lipid accumulation indices and mitochondrial dynamics parameters. Furthermore, in response to the rising consumer demand for higher quality aquatic products driven by improved living standards [32], and basing on that flesh quality is evaluated based on nutritive values, texture, and flavor characteristics [33, 34], this research also examines the paradoxical deterioration of fish flesh quality resulting from hepatic lipid disorders induced by HFD [35]. Specifically, we explore the potential of dietary creatine to mitigate the adverse effects of HFD on muscle flesh quality. The findings of this study are expected to make significant contributions to enhancing the efficiency of HFD utilization and improving lipid metabolism regulation in aquaculture species.

## 2. Materials and Methods

### 2.1. Ethic Statement

All experiments were conducted according to the institutional ethical guidelines of Huazhong Agricultural University (HZAU) on the care and use of experimental animals. Animal research in this study gained approval from the Ethical Committee of Huazhong Agriculture University (identification code: Fish-2023-08-60).

### 2.2. Reagents

Creatine (>99.9% in purity) was purchased from Shanghai Sinopharm Co. Ltd, China and added as the form of creatine hydrate. Tricaine methanesulfonate (MS-222) was from Sigma–Aldrich, MO, US. The kits for analysis of protein concentration, triglyceride (TG), and ATP levels were obtained from Nanjing Jiancheng Bioengineering Institute, China. Trizol reagent for RNA extraction was from Thermo Fisher Science (Waltham, MA, USA, 10296028). Reverse Transcription Kit was from TaKaRa (Tokyo, Japan, 6110A). Other reagents belong to analytical reagents and were from Shanghai Sinopharm Co. Ltd, China.

### 2.3. Juvenile Grass Carp Feeding, Management, and Sample Collection

The experimental diets, as detailed in Table S1, comprised three formulations: a control diet (5.20% lipid, control), a HFD (8.31% lipid, HFD), and a HFD supplemented with 2% creatine (HFD + creatine). These diets were prepared following established protocols from our previous study [36]. Briefly, ingredients were ground, precisely weighed, and thoroughly mixed. The prepared diets were then air-dried and stored at −20°C until utilization.

The feeding trial was conducted at the Fishery Base of Huazhong Agricultural University, adhering to our previously described methodology [36]. Following acclimatization, 270 juvenile grass carp (*C. idella*) with an initial mean body weight of 4.12 ± 0.02 g (mean ± SEM) were randomly distributed into nine circular tanks (300 L capacity), with 30 fish per tank. The fish were fed their respective diets to apparent satiation twice daily (08:30 and 16:30 h) for 8 consecutive weeks. Each dietary treatment was replicated in three tanks. Biometric measurements were conducted at 2-week intervals to monitor growth performance and health status, while mortality was recorded daily. Throughout the experimental period, water quality parameters were maintained as follows: dissolved oxygen 6.53 ± 0.14 mg/L, temperature 28.4 ± 0.3°C, pH 7.73 ± 0.11, and NH_4_-N concentration 0.062 ± 0.003 mg/L.

Upon completion of the 8-week feeding trial, all fish were subjected to a 24-h fasting period prior to sampling. Euthanasia was performed using MS-222 (tricaine methanesulfonate) according to standard protocols. Total biomass was recorded for each tank to calculate growth performance indices, including weight gain (WG), specific growth rate (SGR), and feed conversion ratio (FCR), using the following formulae: WG (%) = (final mean body weight (g) − initial mean body weight (g))/initial mean body weight (g) × 100%; SGR (%/d) = 100%× [ln (final mean body weight (g))-ln (initial mean body weight (g))]/d; FCR = dry feed consumed (g)/wet WG (g). The liver and muscle tissues from six fish were cut, frozen in liquid nitrogen for analysis of enzymatic activities and ATP content. The liver and muscle tissues from another six fish were frozen in the liquid nitrogen for analysis of proximate composition, quantitative real-time PCR analysis (qPCR) and western blot (WB). For biochemical analyses, liver and muscle tissues were collected from 12 fish per treatment group (*n* = 6 for each analysis). Tissue samples from six fish were immediately frozen in liquid nitrogen for subsequent analysis of enzymatic activities and ATP content. The remaining six fish samples were preserved in liquid nitrogen for proximate composition analysis, qPCR, and western blot (WB) analyses.

### 2.4. Sample Analysis

#### 2.4.1. Muscle Proximate Composition

The proximate composition of diet and muscle samples, including moisture, crude lipid, crude protein, and ash contents, was determined following the standard methods of the Association of Official Analytical Chemists [37]. Moisture content was quantified by oven-drying the samples at 105°C until constant weight was achieved. Ash content was analyzed through incineration in a muffle furnace at 550°C for 12 h. Crude protein content was measured using a Kjeltec 8400 Analyzer Unit (FOSS Tecator, Höganäs, Sweden) based on the Kjeldahl method. Crude lipid content was determined by solvent extraction using a Soxtec system HT (FOSS Tecator, Höganäs, Sweden). All analytical procedures were performed in accordance with the methodology described by Yin et al. [38].

#### 2.4.2. Oil Red O, H&E, Bodipy 493/503 Staining, and Transmission Electron Microscopy Observation

Histological examinations were performed using Oil Red O and Hematoxylin & Eosin (H&E) staining techniques, as previously described [39]. For light microscopic analysis, sagittal tissue sections (6–8 μm thickness) were stained with H&E using standard protocols. Additionally, a total of 36 tissue sections were subjected to Oil Red O staining for lipid visualization. All histological images were evaluated using a double-blind assessment protocol to ensure objective analysis.

For cellular-level lipid analysis, Bodipy 493/503 staining was employed to detect lipid droplets (LDs) in hepatocytes, following our established methodology [40]. Fluorescence intensity was quantified using a Leica DMI8 laser scanning confocal microscope. Ultrastructural examination of hepatocytes was conducted through transmission electron microscopy (TEM). Briefly, ultrathin sections were sequentially processed through ethanol dehydration, resin embedding, and dual staining with uranyl acetate and lead citrate prior to TEM observation.

#### 2.4.3. Determination of Triglyceride and ATP Contents, Analysis of FAs β-Oxidation Rate and Cpt1 Activity

The contents of TG were determined by commercial kits (A110-1-1; Nanjing Jiancheng, Nanjing, China) according to the manufacturer's instructions. ATP was measured by using an ATP Colori- metric/Fluorometric Assay Kit (S0026; Beyotime, Nantong, China) according to the manufacturer's instructions.

Analysis of mitochondrial FAs *β*-oxidation rate and carnitine palmitoyl transferase-1 (Cpt1) activity according to our previously published protocol [41]. As for Cpt1 activity assay, mitochondria were isolated from the liver or hepatocytes according to Morash et al. [42]. Cpt1 activity was determined using the method of [43] based on measurement of the initial CoA-SH formation. One unit (IU) of Cpt1 activity was defined as 1 μmol of product formed per min per mg of mitochondrial protein at 25°C.

#### 2.4.4. Analysis of Flavor Nucleotides

The flavor nucleotides assay were determined by published methods [44]. Samples dried to different degrees were weighed, ground sufficiently, and extracted with 10 mL of distilled water. The suspension was boiled for 1 min, cooled and centrifuged at 4500 *g* for 15 min. The residues were washed three times with 10 mL of distilled water, and the combined filtrates were rotary evaporated and redissolved in distilled water to a final volume of 10 mL and filtered through a 0.45 μm micropore filter membrane before analysis.

The assay was performed on a Zorbax Eclipse XDB C18 column (250 × 4.6 mm, 5 μm, Agilent), and the mobile phase was distilled water/methanol/acetic acid/tetrabutylammonium hydroxide (894.5/100/5/0.5, v/v/v/v) with an injection volume of 20 μL at a flow rate of 0.7 mL/min, and the 5′-nucleotides were detected by UV at 254 nm. Each 5′-nucleotide was identified using the authentic 5′-nucleotide (Aladdin Reagent (Shanghai) Co., Ltd, Shanghai, China) and quantified using the calibration curve of the authentic compound against external standards.

#### 2.4.5. Amino Acid Composition Measurements

For analysis of hydrolyzed amino acids according to published methods [45], muscle samples were hydrolyzed in 6 NHCl at 110°C for 24 h. The hydrolysates were evaporated. The remaining materials were dissolved in citric acid buffer solution. Then, samples were analyzed using high-performance liquid chromatography with an hypersil ods column (250 × 4.6 mm, 5 µm) (HPLC, AG1100, Palo Alto, CA, USA). Pre-column derivatization with o-phthalaldehyde and 9-fluorenylmethyl chloroformate was used to identify the FAAs. After standing for 1 h, the mixture was centrifuged at 10,000 × *g* for 10 min. The supernatant was filtered through a 0.22-µm membrane filter and subjected to HPLC.

#### 2.4.6. Analysis of Odorous Compounds

The quantification of odorous compounds, specifically geosmin (GSM) and 2-methylisoborneol (2-MIB), was conducted according to established methodologies [46]. The extraction of these compounds from grass carp muscle tissue was performed using an optimized microwave-assisted distillation system. The distillation apparatus was equipped with a recirculating refrigeration unit maintaining the condenser temperature at 5°C. Microwave parameters were controlled at 120°C with an argon purge flow rate of 25 mL/min. The volatile compounds liberated from the muscle tissue were condensed and collected in glass vials.

Subsequent concentration of GSM and 2-MIB was achieved using a purge and trap (P&T) system (Eclipse 4660, O.I. Analytical Company, USA) comprising a sample concentrator and a #09 adsorption trap. A 5 mL aliquot of distillate was purged with high-purity nitrogen (99.999%) at 40 mL/min for 11 min, with the trap maintained at 20°C during the adsorption phase. Thermal desorption was performed using helium carrier gas (4 min) for direct transfer to the gas chromatography system. Temperature parameters were optimized as follows: water manager at 80°C and trap at 195°C during desorption (pre-desorption at 185°C). System cleaning was achieved through a 10-min bake-out cycle at 240°C (water manager) and 215°C (trap).

Quantitative analysis was performed using gas chromatography-mass spectrometry (GC–MS) in electron ionization (EI) mode. Separation was achieved on an Agilent 19091J-433 capillary column (30 m × 0.25 mm × 0.25 μm) with the injector maintained at 220°C. Helium carrier gas was delivered at 1 mL/min in programmed split mode (3:1 ratio). The oven temperature program initiated at 50°C (1 min hold), followed by a 20°C/min ramp to 240°C, and a final hold at 280°C for 3 min. EI conditions were maintained at 70 eV, with ion source and GC interface temperatures of 230°C and 280°C, respectively. The solvent delay time was set at 5 min to protect the detector.

#### 2.4.7. pH and Water-Holding Capacity Measurements

The muscle pH was determined utilizing a Testo 205 pH meter (Testo AG, Lenzkirch, Germany). The water-holding capacity (WHC) of the samples was assessed through three distinct methodologies: drip loss, flesh leaching loss, and liquid loss, as described in reference [37]. To minimize the influence of muscle heterogeneity on WHC measurements, a standardized protocol was implemented wherein 5 ± 0.5 g of muscle tissue was excised from an anatomically consistent location for each specimen.

#### 2.4.8. Texture Measurements

The textural properties of the muscle samples were quantitatively analyzed using a TA-XT Plus Micro Texture Profile Analyzer (Stable Micro Systems Ltd., Godalming, Surrey, UK) equipped with a flat-bottomed cylindrical probe (P/36R) and a 250 N load cell. The texture profile analysis was conducted in accordance with the standardized methodology previously described by Ma et al. [47]. During analysis, characteristic texture curves were obtained, from which the maximum force (N) was determined as the mean value of triplicate measurements for each sample.

#### 2.4.9. RNA Isolation and Quantitative Real-Time PCR Analysis (qPCR)

Total RNA was extracted using Trizol reagent (Invitrogen, Carlsbad, CA, USA) and subsequently reverse-transcribed into complementary DNA (cDNA) using a Reverse Transcription Kit (Takara, Tokyo, Japan, 6110A). Gene expression analysis was performed through real-time quantitative polymerase chain reaction (qPCR) as previously described [39]. The specific primer sequences utilized in this study are provided in Table S2. Preliminary experiments demonstrated that both *gapdh* and *β-actin* (*M* = 0.27) exhibited the most stable expression patterns among housekeeping genes evaluated. Therefore, relative gene expression levels were normalized to the geometric mean of *gapdh* and *β-actin* expression and quantified using the comparative 2^*−ΔΔCt*^ method.

### 2.5. Transfections and Luciferase Assays

HepG2 cells were cultured in DMEM medium supplemented with 10% (*v*/*v*) heat-inactivated FBS (Gibco/Invitrogen, Carlsbad, CA, USA) in a humidified atmosphere with 5% CO2 at 37°C according to our previous studies [48]. All Plasmids were transiently transfected into HepG2 cells using lipofectamine 2000 (Invitrogen) following the manufacture's protocol. After 24 h incubation, HepG2 cells were harvested to assay the luciferase activity by Dual-Luciferase Reporter Assay System (Promega) following the manufacture's instruction. The relative luciferase activity was presented as the ratio of firefly luciferase to Renilla luciferase. All experiments were performed in triplicates and repeated at least three times.

### 2.6. Western Blot

To identify the protein levels of peroxisome proliferators-activated receptors (Ppar*α*), Cpt1, Mfn2 and dynamin-related protein 1 (Drp1), western blot analysis was performed according to our previous study [39]. In brief, the protein was loaded onto the SDS-PAGE gel and then transferred to the PVDF membrane. Membranes were blocked with 5% skimmed milk and then incubated overnight at 4°C with one of the following primary antibodies: anti-Cpt1*α* (1:1000, A20746; Abclonal, BSN, USA), anti-Ppar*α* (1:1000, 66826-1-Ig; Pro-teintech Group, Wuhan, China), anti-Drp1 (1:1000, 12957-1-AP; Pro-teintech Group, Wuhan, China), anti-Mfn2 (1:1000, 12186-1-AP; Pro-teintech Group, Wuhan, China), anti-Gapdh (1:10000, 60004-1-Ig; Pro-teintech Group, Wuhan, China), respectively. The secondary antibodies were then incubated with the membranes. The membranes were seen by ECL after additional washing. The membranes were seen using enhanced chemiluminescence, and Image J was used to measure the densitometry of these bands.

### 2.7. Statistical Analysis

All data were expressed as mean ± standard error of means (SEM). The normality of data distribution and the homogeneity of variances were analyzed using the Kolmogorov–Smirnov test and Bartlett's test, respectively. Then, data were subjected to one-way ANOVA and Duncan's multiple range test using SPSS 19.0 software, and the minimum significance level was set at *p* < 0.05. Values without the same letter indicated significant difference among different treatments (*p* < 0.05).

## 3. Results

### 3.1. Dietary Creatine Ameliorates the Adverse Effects of HFD on Growth Performance and Feed Utilization

To initially evaluate the ameliorative effects of creatine supplementation on HFD-induced impairments in growth performance and feed utilization in juvenile grass carp, an 8-week feeding trial was conducted. Following the experimental period, survival rates showed no statistically significant differences among the control, HFD, and HFD + creatine groups (Table 1). However, compared to the HFD group, the HFD + creatine group demonstrated a significant improvement in SGR and a marked reduction in feed conversion ratio (FCR) (Table 1). Subsequent analysis of muscle tissue composition revealed no significant differences in crude ash, crude protein, or moisture content among the control, HFD, and HFD + creatine groups (Table 2). Notably, the HFD + creatine group exhibited a significant decrease in muscle crude lipid content compared to the HFD group (Table 2). These findings suggest that dietary creatine supplementation effectively enhances growth performance and feed utilization efficiency while reducing muscle lipid accumulation in juvenile grass carp fed a HFD.

### 3.2. Dietary Creatine Supplementation Enhanced the Flesh Quality in Muscle of Juvenile Grass Carp

The widespread application of HFD has led to a significant decline in the flesh quality of juvenile grass carp, posing a major concern in aquaculture. To address this issue, we investigated the negative impacts of HFD on flesh quality and evaluated the potential ameliorative effects of dietary creatine supplementation on muscle quality in juvenile grass carp. Our findings revealed that HFD significantly increased drip loss, flesh leaching loss, and liquid loss in juvenile grass carp muscle compared to the control group, while muscle pH remained unchanged (Table 3). Conversely, creatine supplementation markedly reduced these parameters in HFD-fed fish (Table 3). These results demonstrate that HFD significantly impairs muscle water-holding capacity, while creatine supplementation effectively mitigates these adverse effects in juvenile grass carp muscle.

Furthermore, texture profile analysis revealed significant improvements in springiness, gumminess, chewiness, and shear force in creatine-supplemented groups compared to HFD-fed fish (Table 4). Although no significant differences were observed in hardness and cohesiveness among the three groups (Table 4), these findings suggest that creatine supplementation alleviates HFD-induced deterioration of muscle texture properties in juvenile grass carp. We also conducted comprehensive analyses of flavor compounds and primary odorants in muscle tissue. HFD significantly reduced the concentrations of flavor nucleotides (AMP and IMP) and flavor amino acids, including umami-taste amino acids (Glu and Asp) and sweet-taste amino acids (Gly and Ala), compared to the control group (Table 5). Additionally, HFD significantly increased the concentrations of key odorant compounds (2-MIB and GSM) in juvenile grass carp muscle (Table 5). Notably, dietary creatine supplementation effectively counteracted these HFD-induced adverse effects on muscle flavor profiles (Table 5). Collectively, these findings demonstrate the protective role of dietary creatine supplementation in mitigating HFD-induced deterioration of flesh quality in juvenile grass carp muscle, highlighting its potential application in aquaculture nutrition.

### 3.3. Dietary Creatine Supplementation Mitigates HFD-Induced Excessive Lipid Accumulation Main via Lipid Catabolism in Liver of Juvenile Grass Carp

**Figure 1:**
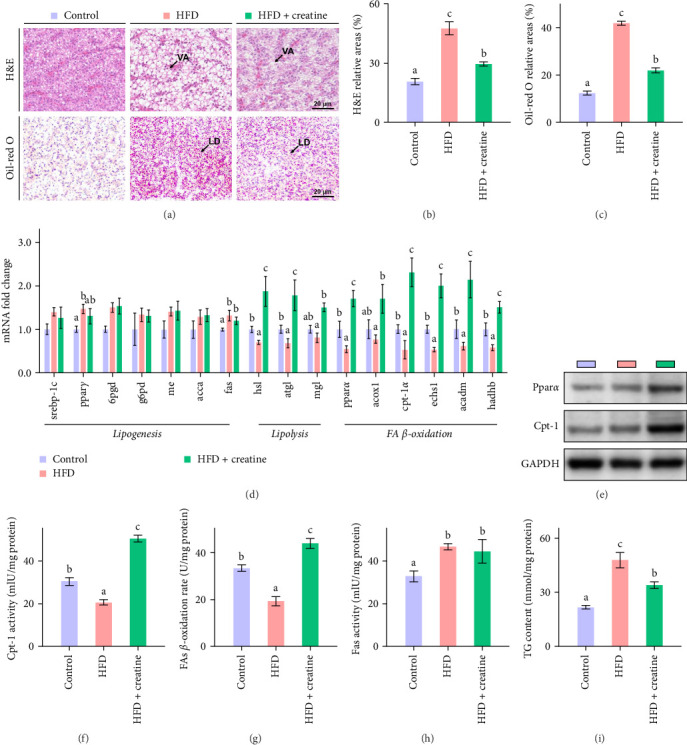
Effects of high-fat diet and dietary creatine on hepatic lipid metabolism of juvenile grass carp. (A) Representative images of liver tissues stained by H&E; 200× magnification, scale bars, 20 μm. (B,C) Relative areas for hepatic vacuoles in H&E staining and LDs in Oil Red O staining. (D) mRNA levels of the genes related to hepatic lipid metabolism. (E) Western blot analysis of Ppar*α* and Cpt-1. (F) Cpt-1 activity. (G) FAs *β*-oxidation rate. (H) Fas activity. (I) TG content. Values are means ± S.E.M. *n* = 3 replicate tanks and were used as three biological replicates. Values without the same letter indicate significant differences among the three treatments (*p* < 0.05).

In the present study, hepatic histopathological analysis revealed that HFD administration resulted in excessive lipid accumulation compared to the control group, as evidenced by histological vacuolization in H&E staining and increased lipid droplet deposition in Oil Red O staining (Figure 1A–C). Notably, dietary creatine supplementation significantly attenuated HFD-induced hepatic steatosis. Mechanistic investigations demonstrated that the protective effect of creatine supplementation was mediated through enhanced lipid catabolism (lipolysis and *β*-oxidation) rather than modulation of lipogenesis.

Quantitative real-time PCR analysis revealed a significant upregulation of key genes involved in lipolysis and *β*-oxidation pathways in the creatine-supplemented group compared to HFD controls, including *hsl*, *atgl*, *mgl*, *pparα*, *acox1*, *cpt1a*, *echs1*, *acadm* and *hadhb* (Figure 1D). These molecular findings were further corroborated by biochemical assays showing enhanced fatty acid *β*-oxidation rates and elevated protein expression levels coupled with increased enzymatic activities of CPT-1 (Figure 1), the rate-limiting enzyme in mitochondrial fatty acid *β*-oxidation [42]. In contrast, no significant alterations were observed in the mRNA expression profiles of lipogenic genes, including *pparγ*, *6pgd*, *me*, *acca* and *fas*, between creatine-supplemented and HFD groups. This observation was further supported by comparable protein expression levels and enzymatic activities of FAS, along with similar TG content measurements (Figure 1E, H, and I). Collectively, these findings demonstrate that dietary creatine supplementation effectively ameliorates HFD-induced hepatic lipid accumulation in juvenile grass carp, primarily through the activation of lipolytic pathways and enhancement of mitochondrial *β*-oxidation capacity, rather than through modulation of de novo lipogenesis.

### 3.4. Dietary Creatine Activates Mitochondrial *β*-Oxidation and Mitigates Hepatic Lipid Accumulation by Mfn2-Mediated Mitochondrial Fusion

**Figure 2:**
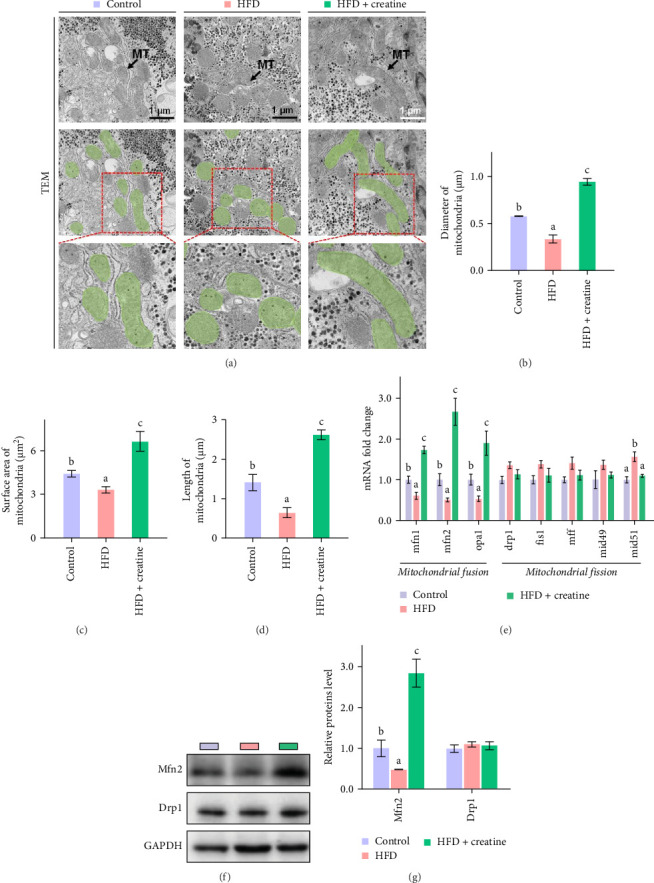
Influences of high-fat diet and dietary creatine on mitochondrial fusion of juvenile grass carp. (A) Representative images of hepatic ultrastructure (TEM), scale bars, 1 μm. (B–D) Quantification of mitochondrial diameter, surface area, and length (*n* = 500–600 mitochondria counted per condition). (E) The mRNA levels of genes involved in mitochondrial fusion. (F,G) Western blot analysis of Mfn2 and Drp1. Values are means ± S.E.M. *n* = 3 replicate tanks and were used as three biological replicates. Values without the same letter indicate significant differences among the three treatments (*p* < 0.05).

First, dietary creatine supplementation demonstrated a protective effect against HFD-induced mitochondrial damage in the liver of juvenile grass carp. TEM analysis revealed significant morphological changes in mitochondria following creatine supplementation, including elongation and increased mitochondrial dimensions (length, diameter, and surface area) (Figure 2A–D). These structural alterations were accompanied by molecular changes in mitochondrial dynamics. Specifically, creatine supplementation upregulated the mRNA expression of key mitochondrial fusion regulators (*mfn1*, *mfn2*, and *opa1*) and increased Mfn2 protein levels. Conversely, the expression of *mid51*, a gene involved in mitochondrial fission, was downregulated (Figure 2). These findings collectively indicate that dietary creatine promotes mitochondrial fusion in the liver of juvenile grass carp.

**Figure 3:**
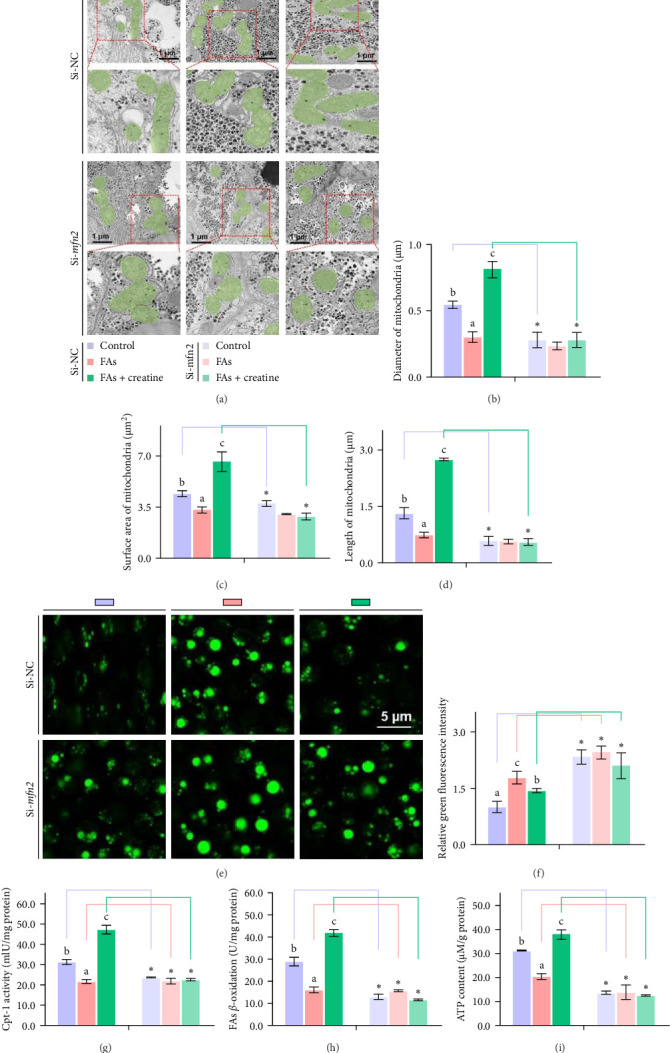
Dietary creatine activates mitochondrial *β*-oxidation and mitigates hepatic lipid accumulation by Mfn2-mediated mitochondrial fusion. (A) Representative images of hepatic ultrastructure (TEM), scale bars, 1 μm. (B–D) Quantification of mitochondrial diameter, surface area and length (*n* = 500–600 mitochondria counted per condition). (E,F) Representative images and relative fluorescence intensity for hepatocytes stained by Bodipy 493/503, scale bars, 5 μm. (G) Cpt1 activity. (H) FAs *β*-oxidation rate. (I) ATP content. All data were expressed as mean ± S.E.M. (*n* = 3 independent biological experiments). Values without the same letter indicate significant differences among the three treatments (*p* < 0.05); asterisks*⁣*_*∗*_indicate significant differences between si-NC and si- *mfn2* groups under the same incubation treatment.

Second, the mechanistic role of creatine in mitochondrial function and lipid metabolism was further investigated in hepatocytes. TEM analysis demonstrated that the creatine-induced increases in mitochondrial dimensions were attenuated by *mfn2* silencing (si-*mfn2*), indicating the essential role of Mfn2 in creatine-mediated mitochondrial fusion (Figure 3A–D). The inhibition of mitochondrial fusion through *mfn2* knockdown exacerbated hepatic LD accumulation (Figure 3E,F) and impaired mitochondrial function, as evidenced by reduced Cpt1 activity, decreased FA *β*-oxidation rate, and lower ATP content. These results demonstrate that creatine supplementation activates mitochondrial *β*-oxidation and reduces hepatic lipid accumulation through Mfn2-mediated mitochondrial fusion in juvenile grass carp hepatocytes.

### 3.5. Dietary Creatine Promotes Mitochondrial Fusion by Activating the Pparα Binding Sites of the Mfn2 Promoter

**Figure 4:**
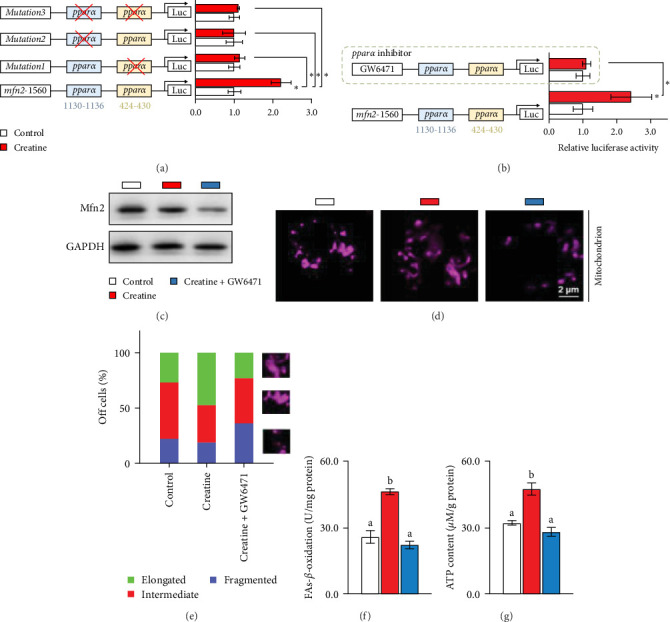
Dietary creatine promotes mitochondrial fusion by activating the *pparα* binding sites of the Mfn2 promoter. (A) Site-mutation assay of *pparα*-binding sites on mfn2-1560 vector in 293T cells. (B) Site-mutation assay with *pparα* antagonists (GW 6471) in 293T cells. (C) Western blot analysis of Mfn2. (D) Confocal images of mitochondria, scale bars, 2 µm. (E) Quantification of the primary hepatocytes from yellow catfish with different states for mitochondria (fragmented, intermediate, or elongated, *n* = 300 cells counted per condition) in pannel D. (F) FAs *β*-oxidation rate. (G) ATP content. Values without the same letter indicate significant differences among the three treatments (*p* < 0.05); asterisks*⁣*_*∗*_indicate significant differences between site-mutation groups under the same incubation treatment.

Next, we further explore the mechanisms by which dietary creatine activates Mfn2-mediated mitochondrial fusion. Here, dual-luciferase reporter assay shown creatine upregulated Mfn2 expression main by activating the two *cis*-acting elements of *pparα* in Mfn2 promoter, which are confirmed by the result from site-mutation assay of *pparα* binding sites after both single-site and double-site mutant (Figure 4A). Further, *pparα* antagonist treatment also have similar results (Figure 4B). Importantly, furthermore, *pparα* antagonist treatment also eradicates creatine-activated Mfn2 protein expression and the function on mitochondrial fusion and *β*-oxidation (Figure 4), further indicating that creatine activates Mfn2 by the *cis*-acting elements of *pparα* in Mfn2 promoter. These findings provide the initial molecular evidence for creatine-activated mitochondrial fusion.

## 4. Discussion

In the aquaculture industry, HFD are extensively utilized to maximize economic benefits. However, there is growing concern regarding the detrimental effects of HFD on farmed fish [49]. Consequently, the development of effective nutritional strategies to mitigate the adverse impacts of HFDs on fish is of paramount importance. Creatine, recognized as a promising green feed additive, has demonstrated potential in enhancing growth performance and physical capabilities in farmed fish [31]. Additionally, creatine has been shown to improve mitochondrial function and reduce lipid accumulation. Previous studies have indicated that creatine plays a role in enhancing lipid metabolism [15, 19]. Nevertheless, the mechanisms by which creatine contributes to growth performance and whether dietary creatine supplementation can alleviate HFD-induced adverse effects in fish remain poorly understood. In farmed fish, the liver and muscle are critical organs and tissues that regulate metabolic homeostasis and growth performance. Therefore, in this study, we formulated diets with HFD and creatine supplementation to assess the mitigative effects of dietary creatine on HFD-induced adverse impacts on growth performance, hepatic lipid metabolism, and muscle quality in juvenile grass carp. This study provides the first evidence of creatine ameliorating HFD-induced adverse effects in farmed fish, offering a comprehensive perspective on multi-organ/tissue interactions and mitochondrial dynamics.

Creatine has emerged as a promising feed additive in aquaculture, particularly for fish fed with low-fishmeal diets. Our current study demonstrates that dietary creatine supplementation mitigates the adverse effects induced by HFD on growth performance. Initially, we observed that HFD significantly suppressed WG and SGR, indicating detrimental effects on the growth performance of juvenile grass carp. These findings align with previous studies [49, 50]. Importantly, creatine supplementation reversed the HFD-induced suppression of WG and SGR in juvenile grass carp. These results are consistent with a study on tilapia (*Oreochromis mossambicus*), which reported that dietary creatine supplementation enhanced the growth performance of farmed fish [51].

The extensive utilization of HFD in aquaculture has garnered significant attention due to their association with excessive hepatic lipid deposition [12, 49], a condition that can lead to substantial mortality rates in fish populations. Consistent with previous studies [49, 52], our experimental results demonstrated that HFD administration indeed induced excessive lipid accumulation in hepatic tissues. Notably, our investigation revealed that dietary creatine supplementation effectively mitigated HFD-induced hepatic lipid deposition. This finding aligns with the work of Tian et al. [22], who reported that creatine supplementation attenuated HFD-induced body WG and hepatic TG accumulation. The pathogenesis of hepatic lipid accumulation primarily stems from an imbalance between lipogenesis and lipid catabolism processes, including lipolysis and mitochondrial *β*-oxidation. While our current study did not observe significant ameliorative effects of creatine supplementation on HFD-induced hepatic lipogenesis, we identified a substantial activation of hepatic lipolysis and *β*-oxidation pathways. These novel findings provide the first evidence that the enhancement of lipolysis, and *β*-oxidation represents the primary mechanism through which dietary creatine ameliorates HFD-induced excessive hepatic lipid deposition.

Given that the mitochondrial matrix serves as the primary site for FAs *β*-oxidation [23], and mitochondrial dynamics play a crucial role in maintaining mitochondrial integrity, homeostasis, and function [25, 26], it is of significant interest to explore the effects of dietary creatine on mitochondrial dynamics, particularly the processes of fusion and fission, and their subsequent impact on FA *β*-oxidation. Our findings indicate that cells in the creatine diet group predominantly exhibited highly elongated and interconnected mitochondria, suggesting an activation of mitochondrial fusion and an inhibition of fission. Further investigation revealed that the expression of Mfn2 was the most prominent phenotype. Recent studies on mitochondrial fusion proteins have demonstrated that Mfn2 is more versatile and exhibits higher membrane tethering efficiency compared to mitofusin 1 (Mfn1) [53]. Previous research has also documented the diverse functions of mitochondrial fusion proteins in adapting to various metabolic states [54]. Therefore, our findings suggest that Mfn2 may be the primary factor responsible for promoting mitochondrial fusion in response to dietary creatine. This result aligns with a study on mouse adipocytes, which demonstrated that creatine can alleviate lipid accumulation through enhanced energy metabolism [19]. In our in vitro study, inhibition of Mfn2 blocked dietary creatine-activated Cpt1 activity and FA *β*-oxidation, leading to impaired ATP content. This represents the first demonstration that dietary creatine activates hepatic FA *β*-oxidation through Mfn2-mediated mitochondrial fusion. Furthermore, our study provides additional molecular insights into how the creatine diet activates the binding sites of the *pparα* transcription factor on the Mfn2 gene promoter, as evidenced by both site-mutation assays and the use of *pparα* antagonists. This is the first molecular evidence that a creatine diet can enhance mitochondrial fusion, although the beneficial role of dietary creatine in mitochondrial function has been well-established.

The enhancement of flesh quality in farmed fish has become a critical concern in aquaculture, driven by consumer demand for superior aquatic products that extend beyond mere increases in yield [55]. In this study, we observed that a HFD adversely affected the flesh quality of grass carp, but these detrimental effects were significantly ameliorated by creatine supplementation, particularly in terms of texture properties. Texture, a vital sensory attribute of fish flesh, encompasses parameters such as firmness, tenderness, chewiness, adhesiveness, and resilience [18]. While limited research has explored the impact of dietary creatine on fish flesh texture, our findings indicate that creatine supplementation enhanced several texture properties, including springiness, gumminess, chewiness, and shear force. High-quality fish muscle is typically characterized by a firm and cohesive texture [18], leading us to conclude that dietary creatine supplementation improved the flesh texture of grass carp. However, Denise et al. [18]. reported no significant changes in texture properties in European seabass (*Dicentrarchus labrax*) following creatine supplementation. This discrepancy suggests that the beneficial effects of creatine on flesh texture may be species specific, warranting further investigation to elucidate the underlying mechanisms.

Flavor is a crucial indicator for evaluating fish flesh quality, primarily determined by flavor, nucleotides, and flavor amino acids, including umami and sweet-taste amino acids [56]. The present study demonstrated that a HFD significantly reduced the flesh flavor of juvenile grass carp, whereas creatine supplementation effectively enhanced it. This improvement was supported by observed changes in the deposition of flavor nucleotides and amino acids in muscle tissue. One potential mechanism for the increased deposition of amino acids is that creatine supplementation may preserve glycine by reducing endogenous creatine synthesis [17, 57]. Additionally, creatine supplementation might decrease the consumption of energy-supplying amino acids, as creatine serves as a critical energy source in the muscle [58]. However, further research is required to elucidate the underlying mechanisms by which creatine influences the deposition of flavor nucleotides. Furthermore, odor is a detrimental factor in fish flesh quality, primarily attributed to two key odorants: 2-MIB and GSM [59]. Interestingly, this study revealed that creatine supplementation significantly reduced the levels of these odorant compounds (2-MIB and GSM) in grass carp muscle. The deposition of 2-MIB and GSM is closely associated with tissue lipid content [59]. Given that creatine supplementation was found to reduce hepatic lipid content by activating mitochondrial fatty acid *β*-oxidation, we hypothesize that the observed decrease in 2-MIB and GSM deposition may result from the overall reduction in lipid content in juvenile grass carp. Further studies are warranted to validate this hypothesis and explore the detailed mechanisms involved.

This present study serves as a continuation of the previous research [44], and both these two studies aim to elucidate the mechanisms by which creatine promotes the growth performance and feed utilization efficiency of grass carp, thereby exploring the potential application of creatine as a feed additive in aquaculture. However, there are fundamental differences between the two studies. Given the widespread use of HFD in aquaculture and the growing industry concern over their negative impacts, from the perspective of hepatic lipid metabolism, this present study builds upon the previous research by further investigating the role of creatine in alleviating hepatic lipid disorders and the decline in muscle quality induced by HFD. Additionally, this study identified that MFN2-mediated mitochondrial fusion plays a significant role in this process, which represents another key differentiating finding from the earlier research.

## 5. Conclusions

**Figure 5:**
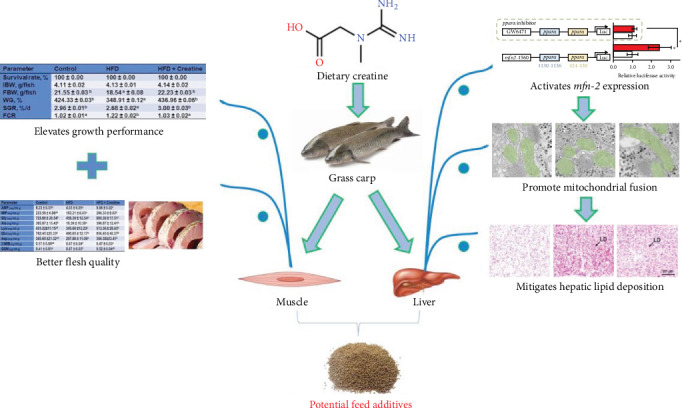
Graphical conclusions for creatine ameliorate the adverse effects of HFD on muscle flesh quality, hepatic lipid metabolism *via* activating Mfn2-mediated mitochondrial fusion in juvenile grass carp.

The present investigation evaluated the impact of creatine supplementation on growth performance and feed utilization in juvenile grass carp through an 8-week feeding trial involving a HFD and dietary creatine supplementation. Notably, creatine supplementation significantly mitigated HFD-induced adverse effects on hepatic lipid metabolism by activating Mfn2-mediated mitochondrial fusion in juvenile grass carp. Furthermore, we identified that the binding sites of the *pparα* transcription factor on the Mfn2 gene promoter constitute the primary mechanism through which creatine activates mitochondrial fusion. Additionally, creatine supplementation ameliorated HFD-induced detrimental effects on the flesh quality of juvenile grass carp. From a comprehensive multiorgan/tissue and mitochondrial dynamics perspective, this study offers a viable approach for developing effective nutritional strategies to alleviate HFD-induced adverse effects in farmed fish. A schematic representation of the proposed mechanism is illustrated in Figure 5.

## Figures and Tables

**Table 1:** Effect of dietary creatine supplementation on growth performance, feed utilization, and morphological parameters of juvenile grass carp fed high-fat diets.

Parameter	Control	HFD	HFD + creatine
Survival rate, (%)	100.00 ± 0.00	100.00 ± 0.00	100.00 ± 0.00
IBW, g/fish	4.11 ± 0.02	4.13 ± 0.01	4.14 ± 0.02
FBW, g/fish	21.55 ± 0.03^b^	18.54^a^ ± 0.08	22.23 ± 0.03 ^b^
WG, (%)	424.33 ± 0.03^b^	348.91 ± 0.12^a^	436.96 ± 0.06^b^
SGR, (%)/d	2.96 ± 0.01^b^	2.68 ± 0.02^a^	3.00 ± 0.03^b^
FCR	1.02 ± 0.01^a^	1.22 ± 0.02^b^	1.03 ± 0.02^a^

*Note:* All data were expressed as mean ± S.E.M. (*n* = 3), *p*  < 0.05. Values without the same letter indicate significant difference among different treatments. IBW (g/fish), initial mean body weight; FBW (g/fish), final mean body weight; WG (weight gain, %) = (FBW-IBW)/IBW × 100%; SGR (specific growth ratio, (%) /d) = 100%× [ln (FBW)-ln (IBW)]/d; FCR (feed conversion ratio) = dry feed consumed (g)/wet weight gain (g).

**Table 2:** Effect of dietary creatine supplementation on common nutritional component contents in muscle of juvenile grass carp fed high-fat diets.

Parameter	Control	HFD	HFD + creatine
Crude ash (%)	1.23 ± 0.11	1.26 ± 0.23	1.14 ± 0.33
Crude protein (%)	17.43 ± 0.46	17.01 ± 0.75	17.51 ± 0.83
Crude lipid (%)	1.22 ± 0.02^a^	1.83 ± 0.24^b^	1.44 ± 0.26^a,b^
Moisture (%)	77.54 ± 0.15	78.25 ± 0.23	77.31 ± 0.45

*Note:* All data were expressed as mean ± S.E.M. (*n* = 3), *p*  < 0.05. Values without the same letter indicate significant difference among different treatments.

**Table 3:** Effect of dietary creatine supplementation on muscle pH and water holding capacity of juvenile grass carp fed high-fat diets.

Parameter	Control	HFD	HFD + creatine
Drip loss	5.66 ± 0.23^a^	7.66 ± 0.52^b^	4.73 ± 0.51^a^
Flesh leaching loss	4.56 ± 0.31^a^	7.65 ± 0.44^b^	4.11 ± 0.51^a^
Liquid loss	12.82 ± 0.65^a,b^	18.44 ± 1.83^b^	11.36 ± 0.33^a^
pH value of muscle	6.31 ± 0.43	6.41 ± 0.44	6.43 ± 0.47

*Note:* All data were expressed as mean ± S.E.M. (*n* = 3), *p*  < 0.05. Values without the same letter indicate significant difference among different treatments.

**Table 4:** Effect of dietary creatine supplementation on muscle texture properties of juvenile grass carp fed high-fat diets.

Parameter	Control	HFD	HFD + creatine
Hardness (g)	2345.12 ± 141.31	2432.36 ± 200.05	2603.87 ± 286.14
Cohesiveness	0.73 ± 0.08	0.80 ± 0.04	0.74 ± 0.07
Springiness (mm)	1.77 ± 0.22^b^	1.13 ± 0.03^a^	1.81 ± 0.13^b^
Gumminess (N)	27.12 ± 2.36^b^	14.34 ± 2.31^a^	38.67 ± 3.91^c^
Chewiness (N)	36.33 ± 2.21^b^	25.23 ± 2.57^a^	52.51 ± 4.61^c^
Shear force	455.23 ± 21.76^b^	331.33 ± 25.78^a^	469.21 ± 32.41^b^

*Note:* All data were expressed as mean ± S.E.M. (*n* = 3), *p*  < 0.05. Values without the same letter indicate significant difference among different treatments.

**Table 5:** Effect of dietary creatine supplementation on flavor nucleotides, amino acids, and key odorants compounds in muscle of juvenile grass carp fed high-fat diets.

Parameter	Control	HFD	HFD + creatine
AMP (mg/100 g)	8.23 ± 0.31^b^	4.03 ± 0.05^a^	9.96 ± 0.02^c^
IMP (mg/100 g)	223.56 ± 6.88^a,b^	163.21 ± 6.03^a^	286.33 ± 8.63^b^
Gly (mg/100 g)	723.86 ± 20.54^b^	456.39 ± 12.54^a^	889.36 ± 17.51^c^
Ala (mg/100 g)	365.87 ± 15.42^b^	16.39 ± 10.38^a^	396.87 ± 12.41^b^
Lys (mg/100 g)	451.22 ± 11.15^ab^	345.69 ± 12.23^a^	512.36 ± 26.63^b^
Glu (mg/100 g)	762.45 ± 25.33^b^	490.65 ± 12.17^a^	856.45 ± 40.57^b^
Asp (mg/100 g)	345.69 ± 21.32^a,b^	297.86 ± 11.09^a^	396.38 ± 23.41^b^
2-MIB (ng/100 g)	0.57 ± 0.06^a,b^	0.87 ± 0.04^b^	0.47 ± 0.03^a^
GSM (ng/100 g)	0.41 ± 0.01^a^	0.87 ± 0.03^b^	0.52 ± 0.04^ab^

*Note:* All data were expressed as mean ± S.E.M. (*n* = 3), *p*  < 0.05. Values without the same letter indicate significant difference among different treatments.

Abbreviations: 2-MIB, 2-methylisoborneol; AMP, adenosine monophosphate; GSM, geosmin; IMP, inosinemonophosphate.

## Data Availability

The data are available from the corresponding author on reasonable request.

## Nomenclature

AMP: Adenosine monophosphate
Asp: Aspartic acid
ATP: Adenosine triphosphate
Cpt1: Carnitine palmitoyl transferase-1
Drp1: Dynamin-related protein 1
DTNB: 5,5-Dithio-bis- (2-nitrobenzoic acid)
Echs1: Enoyl-CoA hydratase
FAs: Fatty acids
FAS: Fatty acid synthase
FBW: Final mean body weight
FCR: Feed conversion rate
GAA: Guanidinoacetic acid
Gapdh: Glyceraldehyde 3-phosphate dehydrogenase
Glu: Glutamate
GSM: Geosmin
HFD: High-fat diet
H&E: Hematoxylin and eosin
IMP: Inosinemonphosphate
LDs: Lipid droplets
Mfn1: Mitofusin 1
Mfn2: Mitofusin 2
Mgl: Monoglycerolase
MS-222: Tricaine methanesulfonate
N-SIM: Nikon Structured Illumination Microscope
NADPH: Nicotinamide adenine dinucleotide phosphate
PA: Palmitoleic acid
PBS: Phosphate-buffered saline
Ppar*α*: Peroxisome proliferators-activated receptors
qPCR: Quantitative real-time PCR analysis
SGR: Specific growth rate
Srebp1: Sterol regulatory element-binding transcription factor 1
TEM: Transmission Electron Microscope
TG: Triglyceride
WG: Weight gain
WB: Western blot
WHC: Water holding capacity
2-MIB: 2-Methylisoborneol
18s rRNA: (Adenine1779-N6/adenine1780-N6)-dimethyltransferase.

## Supporting Information

Supporting Information Feed formulation in this experiment was shown in Table S1. The primer sequences used in this analysis are given in Table S2.
