# Supplementary material for: Creatine Ameliorates the Adverse Effects of High-Fat Diet on Hepatic Lipid Metabolism via Activating Mfn2-Mediated Mitochondrial Fusion in Juvenile Grass Carp
Source: Aquac Nutr. 2025 May 8;2025:1151656. doi: 10.1155/anu/1151656 (PMC12081150; doi:10.1155/anu/1151656)
Supplement: Supporting Information — Feed formulation in this experiment was shown in Table S1. The primer sequences used in this analysis are given in Table S2. [file 1151656.f1.docx]

**SUPPLEMENTAL INFORMATION:**

**Creatine ameliorates the adverse effects of high-fat diet on** **hepatic lipid metabolism *via* activating Mfn2-mediated mitochondrial fusion in juvenile grass carp**

**Nan-Jun Hu^a^, Guang-Li Feng^a^, Xiao-Hong Lai^b^, Peng Mo^b*^, Yu-Feng Song^a*^,**

^a^ *Key Laboratory of Freshwater Animal Breeding, Ministry of Agriculture, Fishery College, Huazhong Agricultural University, Wuhan 430070, China*

^b^ *College of Animal Science and Technology, Jiangxi Agricultural University, Nanchang 330045, China*

***Shortened Title:*** *Creatine improves hepatic lipid metabolism by mitochondrial fusion.*

**^*^*Corresponding author:*** *Prof. Yu-feng Song, Tel.: +86-27-8728-2113; Fax: +86-27-8728-2114; Email address: syf880310@mail.hzau.edu.cn (Y-F. Song); Prof. Peng Mo, Email address:* *pengmowell@jxau.edu.cn.*

***Table S1. Ingredients and proximate analysis of experimental diets.***

| Ingredients (g kg^−1^) | Control | HFD | HFD + Creatine |
| --- | --- | --- | --- |
| Wheat | 180 | 180 | 180 |
| Soybean meal-43% | 250 | 250 | 250 |
| Rapeseed meal-36% | 150 | 150 | 150 |
| Sunflower seed meal | 50 | 50 | 50 |
| Corn DDGS | 150 | 150 | 150 |
| Corn husk | 100 | 70 | 50 |
| Pork powder | 20 | 20 | 20 |
| Rice bran | 31 | 31 | 31 |
| Soybean oil | 30 | 60 | 60 |
| Ca(H_2_PO_3_)_2_·H_2_O | 25 | 25 | 25 |
| Vitamin premix | 2 | 2 | 2 |
| Mineral mixture | 6 | 6 | 6 |
| Lysine | 4 | 4 | 4 |
| Choline | 2 | 2 | 2 |
| Creatine | 0 | 0 | 20 |
| *Proximate analysis (%, dry weight)* | | | |
| Moisture | 9.82 | 9.91 | 10.03 |
| Crude protein | 27.99 | 26.89 | 27.24 |
| Crude lipid | 5.20 | 8.11 | 8.14 |
| Crude ash | 4.31 | 4.24 | 4.15 |

Vitamin premix (mg or IU per kg diet): retinylacetate, 10000 IU; cholecalciferol, 1000 IU; all-rac-a-tocopheryl acetate, 30 IU; menadione nicotinamide bisulfite, 7; thiamine hydrochloride, 6; riboflavin, 3; pyridoxine hydrochloride, 12; D-calcium pantothenate, 30; niacin, 50; biotin, 1; folic acid, 6; cyanocobalamine, 0.03.

Mineral mixture (mg per kg diet): Ca(H_2_PO_3_)_2_·H_2_O, 1000; FeSO_4_·7H_2_O, 40; ZnSO_4_·7H_2_O, 40; MnSO_4_·H_2_O, 40; CuSO_4_·5H_2_O, 2; CaIO_3_·6H_2_O, 3; Na_2_SeO_3_, 0.05; CoSO_4_, 0.05.

***Table S2. Primers used for quantitative real-time PCR analysis and siRNA sequences***

| gene | Forward primer (5’-3’) | Reverse primer (5’-3’) | Accession no. |
| --- | --- | --- | --- |
| *mfn1* | TGACGAGTAGGTGCACGTTT | GTCCTCTTGCTCCATGGCTT | XM_051915574.1 |
| *mfn2* | TGTAGCCGGAGGAAAGTAGCA | GAGACATTGCCCAGGGAAGAA | XM_051901881.1 |
| *opa1* | TCCTGCTTCTTTACGGGTTCC | CGCTCCAATGATCAGAGCCT | XM_051896998.1 |
| *fis1* | GACTTGTCCGTGTTCTCACTTTG | GTTCTCGTCCCAGTGTGATAGTT | XM_051899406.1 |
| *mff* | CTGCTGATAGCCGGAATGATAGT | AGAAAGACAGGACAGGACGTTAC | XM_051877607.1 |
| *mid49* | TAACACTGAGCAGAACTGATCCC | GATTTAAGAACAGCCCACACCAC | XM_051889926.1 |
| *mid51* | CATCGCTGCTTGTTGATCTCG | TCAAGCCGAGAATCAGCCTT | XM_051886558.1 |
| *Srebp-1c* | TCACCAATCCTGACCACCTC | GATGCAAGGTGACGCTACTG | KJ162572.1 |
| *pparγ* | ATTTATGCTGCACAGGCGGT | AGTTCGTCCAGTTCCAGAGC | EU847421 |
| *6pgd* | TGGAATACGGCACACCTGTC | TACTGGCCTGAACCCTCTCA | KP148259 |
| *me* | TCTGAGAGAGGTCATCCCCA | GGCCGCAATCAGACAGTTAG | XM_051867139 |
| *acca* | GGGCACAAAGACCGACAGAT | GGCCTGGAAGCGTTTAGACT | GU908474 |
| *fas* | AGGTCGCCTTCCTGAGTCTA | GTTTCCACCGTCTGTCGTCT | GQ466046 |
| *hsl* | CGCAGTTCATTGAGTGACAGTTT | CACCCCCTTCAGACGATCAG | FJ843081 |
| *atgl* | CTACAGGGGGCATGTCCAAG | GCAGCACCCACTCTTTTGTG | HQ845211 |
| *mgl* | TGTCGTATTGCAGTGGGTCT | ACTTTTCTGCCTCCTCGTGA | XM_051907891.1 |
| *pparα* | ACTGCTGAACAGCGCAAAAA | TCTTGGGTGGAAGGGTCGTA | FJ231987 |
| *acox1* | ACTGCTGAACAGCGCAAAAA | TCTTGGGTGGAAGGGTCGTA | XM_051895864.1 |
| *cpt-1α* | GCGCACGGATCAGCTATTG | CGTTCTGCCTGCTGCAAAAA | JF728839 |
| *echs1* | TTACCGTTGACCTCGCACAA | ACTGCATTGCCTTGTTGCTG | XM_051918081.1 |
| *acadm* | GTCGTTGATTGGTTTGCGCT | CTGAGTCCAGTCCGAACACC | MW713128 |
| *hadhb* | CCACCATGGAACAGATGGCT | GCACCGTCAGTCAGGAAAGA | MW713132 |
| *gapdh* | ACCACTAACTGCTTGGCTCC | GTCTTCTGTGTGGCGGTGTA | AF544974 |
| *rpl7* | AAGGCCGTGCGTCTAAAGAA | TTGCGAGCCATCCTGTTCAT | XM_051865923.1 |
| *18s rRNA* | ATTGGAGGGCAAGTCTGGTG | CCCGAGATCCAACTACGAGC | XR_007928648.1 |
| *elfa* | CAGGAAACCCCTCTATCAGCA | TCGAGTTGAACTTCTCCGCC | XM_051861094 |
| *hprt* | TGGTGCCTTAGATGGTAAATTTGT | TGGCTGAACAATGGTGAGGT | MW713133 |
| *ubce* | TACGTGTTTGAGAGTCGTTTGT | CTCTCAGAGCGGAAGTGCTG | XM_051872265.1 |
| *tuba* | TCGACTCCATCGAGGGTGAA | TGTGTCCCTAACATTTAGGAGTAG | MW713135 |
| siRNA sequences | | | |
| *si-mfn2* | CCGAAUCUUCUUUGUUUCUGCUAAA | UUUAGCAGAAACAAAGAAGAUUCGG |  |
